# Glyceraldehyde-3-phosphate dehydrogenase (GAPDH) interaction with 3' ends of Japanese encephalitis virus RNA and colocalization with the viral NS5 protein

**DOI:** 10.1186/1423-0127-16-40

**Published:** 2009-04-15

**Authors:** Shang-Hua Yang, Mei-Lan Liu, Chih-Feng Tien, Shih-Jie Chou, Ruey-Yi Chang

**Affiliations:** 1 Institute of Biotechnology and Department of Life Science, National Dong Hwa University, Taiwan, ROC

## Abstract

Replication of the Japanese encephalitis virus (JEV) genome depends on host factors for successfully completing their life cycles; to do this, host factors have been recruited and/or relocated to the site of viral replication. Glyceraldehyde-3-phosphate dehydrogenase (GAPDH), a cellular metabolic protein, was found to colocalize with viral RNA-dependent RNA polymerase (NS5) in JEV-infected cells. Subcellular fractionation further indicated that GAPDH remained relatively constant in the cytosol, while increasing at 12 to 24 hours postinfection (hpi) and decreasing at 36 hpi in the nuclear fraction of infected cells. In contrast, the redistribution patterns of GAPDH were not observed in the uninfected cells. Co-immunoprecipitation of GAPDH and JEV NS5 protein revealed no direct protein-protein interaction; instead, GAPDH binds to the 3' termini of plus- and minus-strand RNAs of JEV by electrophoretic mobility shift assays. Accordingly, GAPDH binds to the minus strand more efficiently than to the plus strand of JEV RNAs. This study highlights the findings that infection of JEV changes subcellular localization of GAPDH suggesting that this metabolic enzyme may play a role in JEV replication.

## Background

Japanese encephalitis virus (JEV) is a mosquito-borne flavivirus that causes acute encephalitis in humans, with a high fatality rate of 20 to 50% [1]. It contains a single-stranded positive RNA genome of 10,976 nucleotides (nts) in length that encodes a single large open reading frame (ORF) flanked by a 5'-untranslated region (5'-UTR, 95 nts) and a 3'-untranslated region (3'-UTR, 585 nts). The ORF is translated as a single polyprotein that undergoes co- and post-translational processing to yield three structural (C, prM, and E), and seven nonstructural proteins (NS1, NS2A, NS2B, NS3, NS4A, NS4B, and NS5) [2].

The largest viral protein, NS5, functions as an RNA-dependent RNA polymerase (RdRp) and plays a major role in amplification of viral RNAs [3]. It has been reported that the NS5 protein binds to the 3' end stemloop (SL) RNA as well as associates with NS3 in the replication complex (RC) [4]. Replication of flavivirus RNA takes place in the cytoplasm by an asymmetric and semiconservative mode resulting in 10 to 100-fold greater plus strands compared to minus strands [5]. However, the major replicase proteins NS3 and NS5 were also found to localize within the nucleus [6,7]. The reason for nuclear localization of these viral proteins remains unknown; viral proteins may decoy some host factors for assisting viral replication. Numerous studies have shown that replication of RNA viruses is involved in many specific RNA-RNA, RNA-protein, and protein-protein interactions. Host factors contribute to various steps in these interactions including translocation of viral RNA and proteins, stabilizing/assembly of RC, and modulation of viral enzymes [8]. In order to obtain more detailed information regarding the interaction between host proteins and the defined viral nucleic acid and/or proteins, we investigated the host factors associating with the main replicase enzyme, NS5, in the JEV-infected cells using yeast-two hybrid screening. Several host factors were found and further characterized by coimmunoprecipitation (co-ip) and immunofluorescence assays (IFA) (unpublished data). The initial observation of GAPDH colocalized with JEV NS5 was to use it as an endogenous control for immunofluorescence assays (IFA) because its constant expression has been frequently used as an internal control in many studies. To our surprise, GAPDH was found to be colocalized with JEV NS5 by IFA. It should be noted that GAPDH was not identified by the yeast two-hybrid assay.

GAPDH is a key glycolytic enzyme that plays a pivotal role in energy production [9]. During the past twenty years, however, numerous studies indicated that GAPHD is a multifunction protein in addition to its traditional role in glycolysis [10]. The enzyme has been found to play many roles including membrane fusion, DNA replication/repair, RNA transport [9,11], apoptosis [12,13], oxidative stress [14], and cytoskeleton assembly [15,16]. Many of these new functions require GAPDH to be associated into a series of multienzyme complexes and correlated with subcellular localization. Indeed, GAPDH is present in both cytoplasm and nucleus indicating that it may shuttle between the two compartments. The nuclear-GAPDH appears to be involved in the initiation of one or more apoptotic cascades [17], play a role in DNA transcription/replication [18], and assist in maintenance of telomeres [19].

In this study, we demonstrate that the colocalization of GAPDH with NS5 in JEV-infected cells was *via *binding to viral RNAs rather than binding to the NS5 protein directly. In addition, GAPDH binds to the minus strand more efficiently than to the plus strand of JEV RNAs. The subcellular localization of GAPDH changed upon JEV infection suggesting that GAPDH may play a role during the JEV life cycle.

## Materials and methods

### Cells and viruses

Baby hamster kidney (BHK-21) cells were grown in RPMI 1640 medium supplemented with 2% fetal bovine serum (FBS) (Gibco-BRL) at 37°C. Human embryonic kidney (HEK293) cells were grown at 37°C in Dulbecco's Modified Eagle's Medium (DMEM) supplemented with 8% FBS. Cells were infected with the JEV RP9 strain [20] at 1 PFU/cell and incubated 1 h for virus adsorption for all experiments.

### Immunofluorescence analysis

Cells grown on coverslips were either uninfected or infected with JEV. At indicated time points postinfection, cells were washed with phosphate-buffered saline followed by fixation with ice-cold methanol. The fixed cells were then incubated with the rabbit anti-NS5 antibody or the mouse anti-GAPDH antibody (Novus). For double labeling of NS5 and GAPDH, the cells were washed and incubated with FITC-conjugated goat anti-rabbit and CY3-conjugated donkey anti-mouse secondary antibodies (Jackson). The coverslips were finally washed, mounted, and examined using a confocal microscope (Leica TCS SL) with 1000× magnification.

### Nuclear/Cytosol fractionations

The separation of nuclear and cytoplasmic extract was done with the Nuclear/Cytosol Fractionation Kit according to the manufacturer's protocol (BioVision). Fractionation was performed at 6-h intervals of postinfection, and the uninfected controls were done simultaneously. Fifteen μg of protein was used per lane and separated on a SDS-10% polyacrylamide gel for Western blot analysis. After blotting, the PVDF membrane was cut horizontally into three pieces: (i) the top piece with estimated molecular weight above 72 kDa was probed with Rabbit anti-NS5 antibody, (ii) the middle piece with molecular weight between 41 and 72 kDa was probed with mouse anti-β-actin antibody (Sigma), and (iii) the bottom piece with protein mass smaller than 41 kDa was probed with Mouse anti-GAPDH antibody (Novus), respectively. Equal amount of proteins was used on separated gel for blotting with Rabbit anti-β-tubulin antibody (Novus) as a cytoplasmic specific subcellular marker. The membranes were incubated with goat anti-rabbit or rabbit anti-mouse IgG coupled with horseradish peroxidase (Jackson) as standard procedure. Signals were revealed using a chemiluminescence kit (ECL, Amersham Parmacia Biotech), visualized using a luminescent image analyzer (LAS-3000, Fujifilm) and analyzed using Multi Gauge software (Fujifilm).

### Co-immunoprecipitations

Cells (1 × 10^7 ^cells) were harvested at 24 hpi in RIPA buffer (150 mM NaCl, 1% IGEPAL CA-630, 0.5% sodium deoxycholate, 0.1% SDS, 50 mM Tris-HCl, pH 8.0, 1 mM phenylmethylsulfonyl fluoride). Cell lysates were incubated on ice for 10 min and cell debris was removed by centrifugation. One milligram of whole cell extract per reaction was precleaned with 20 μl protein G-plus agarose beads (Santa Cruz) at 4°C for 1 hour with rotation. The clarified supernatants were incubated with rabbit anti-NS5 antibody, anti-NS3 antibody, anti-hemagglutinin (HA) (LAB Vision), or anti-GAPDH antibody (AbFrontier) at 4°C overnight. Protein G-plus agarose beads were added to the mixture and incubated for 1 hour at 4°C to pull down antibody-specific complexes, and the precipitate was washed with 1 ml of RIPA buffer five times. The final precipitate was then boiled in protein loading buffer for 5 min and ran on SDS-10% PAGE for Western blot analysis.

### Plasmid construction

Two plasmids that harbor a T7 promoter directing the synthesis of 3'-ends of plus- and minus-strands of JEV RNAs were constructed by PCR. JEV specific cDNA was made as previously described [21]. pUC18JEV(+)3'SL was constructed with the PCR products amplified using the T7 promoter (lowercase) containing primer (5'-taatacgactcactataGGGAGATCTTCTGCTCTATC-3', corresponding to JEV nt 10890–10909) and the *Sal *I site-containing primer (5'-ACG*CGTCGAC*AGATCCTGTGTTCTTCCTCACCACCAG-3', complementary to nt 10950 to 10976). pUC18JEV(-)1–160 was constructed with the PCR products amplified using the *Xba *I site-containing primer (5'-GC*TCTAG*AGAAGTTTATCTGTGTGAACTTCTTGGCTTAG-3', corresponding to nt 1–32) and the T7-containing primer 5'-taatacgactcactataGGTAGGCCGCGTTTCAGCATATTG-3' (complementary to nt 137–160). All clones were confirmed by commercial sequencing.

### Electrophoretic-Mobility shift assay (EMSA)

Recombinant plasmids, pUC18JEV(+)3'SL and pUC18JEV(-)1–160, were linearized with *Sal *I and *Xba *I, respectively. Radioactively labeled riboprobes were generated by run-off transcription in presence of α-^32^P-UTP (3000 Ci/mmol) and gel purified. Approximately 10^5 ^cpm of riboprobe per reaction was heat-denatured at 85°C for 5 min and equal volume 2× renaturing buffer (40 mM HEPES, pH 7.9, 120 mM KCl, 4 mM MgCl_2_) and 1 μl Ribonuclease inhibitor (40 U/μl, Takara) were added and incubated at 45°C for 30 mins. The riboprobe with 50 ng to 1 μg of GAPDH (Sigma), or 1 μg of Bovine Serum albumin (BSA), was incubated at room temperature for 30 mins for complex formation. One μl of Heparin (1 μg/μl) was added to the mixture and incubated for 10 mins. The RNA-protein complexes (RPC) were resolved by electrophoresis through a 4% polyacrylamide gel in 0.5× TBE and analyzed by autoradiography.

## Results

### Colocalization of GAPDH with JEV NS5

**Figure 1:**
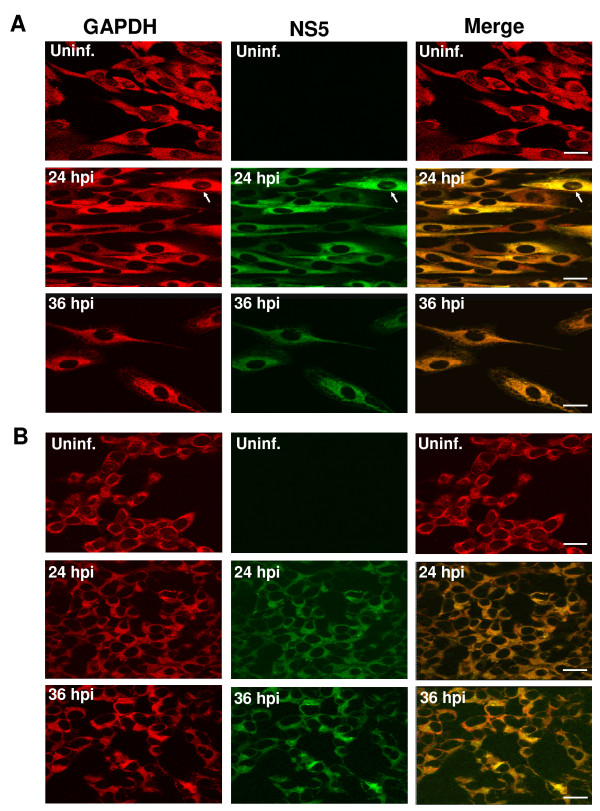
**GAPDH and NS5 proteins colocalize within the JEV-infected cells**. Double staining of GAPDH (red) and NS5 (green) proteins in BHK-21 (A) or HEK293 cells (B) was done as described in Materials and Methods. Cells were stained as uninfected (Uninf.) or at hours postinfection (hpi). Colocalization of both NS5 and GAPDH was evidenced by merged images (yellow). The scale bar is 40 μM.

To investigate the interaction of JEV NS5 with host factor GAPDH in JEV-infected BHK-21 and HEK293 cells, we use immunofluorescent staining. As expected, GAPDH (red color) was detected in the cytoplasm as well as in the nucleus of the uninfected cells (Fig. 1A). In contrast, the GAPDH was shown mainly in the cytoplasm of JEV-infected BHK-21 cells at 24 hpi, while only a few GAPDH were observed in the nucleus. Notably, not any GAPDH was detected in the nucleus of JEV-infected BHK-21 cells at 36 hpi (Fig. 1A), suggesting that infection of JEV may cause redistribution of the nucleus-localized portion of GAPDH to the cytosol. Positive staining of the NS5 proteins (green) were predominantly detected in the cytoplasm throughout the infection period whereas only a few proteins were observed in the nucleus of JEV-infected BHK-21 cells at 24 hpi (Fig. 1A, white arrows). The merged images showed that GAPDH and NS5 protein colocalized in the cytoplasm throughout the infection period and in the nucleus of a few cells at 24 hpi (Fig. 1A). A similar colocalization pattern was also observed in JEV-infected HEK293 cells, except for the cells being smaller than BHK-21 cells (Fig. 1B). Overall, the redistribution of the nucleus-localized portion of GAPDH to cytosol in JEV-infected cells and colocalization with NS5 suggest that GAPDH may play a role in JEV infection.

### Subcellular localization of GAPDH after JEV infection

**Figure 2:**
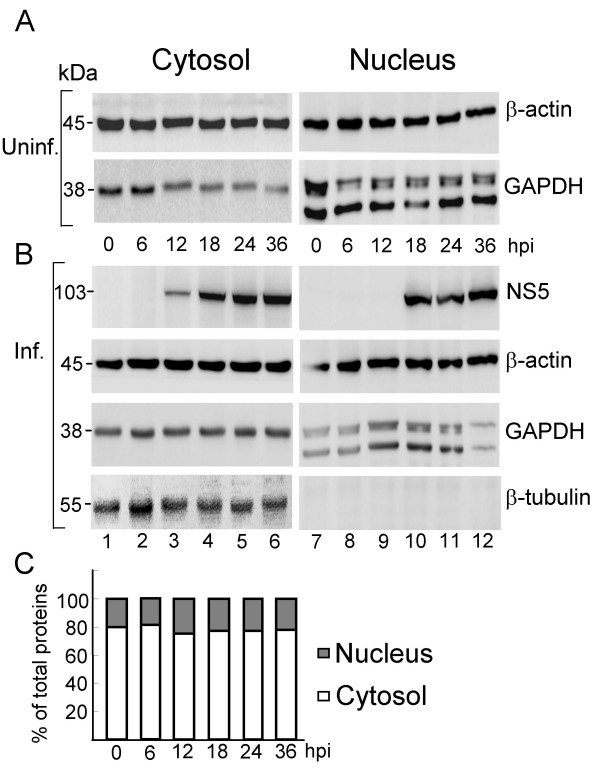
**Evidence of GAPDH redistribution in JEV-infected HEK293 cells**. Proteins were extracted from uninfected cells (A) at a time simultaneous with those hours postinfection (hpi) with JEV (B). Cytoplasmic (lanes 1 to 6) and nuclear proteins (lanes 7 to 12) were fractionated as described in Materials and Methods. Western blots were done separately with antibodies as indicated on the right. (C). Relative amounts of proteins isolated from cytosolic or nuclear fraction during each time point, yet equal amounts (15 μg) of proteins were loaded per lane on panels A and B.

To further investigate the redistribution of GAPDH from the nucleus to the cytoplasm after JEV infection, we perform subcellular fractionation of nucleus and cytoplasm from both uninfected and infected cells, followed by Western blot analyses. As shown in Fig 2A., total amounts of the cytoplasmic-GAPDH (lanes 1 to 6) and the nuclear-GAPDH (lanes 7 to 12) are relatively constant at each time point in the uninfected cells. In contrast, in the fraction of infected cells, cytoplasmic-GAPDH remains constant (Fig 2B, lanes 1 to 6), while the nuclear-GAPDH increasing at 12 to 24 hours postinfection (hpi) and decreasing at 36 hpi were observed (Fig 2B, lanes 7 to 12) indicating that the nucleus-localized portion of GAPDH redistributed after viral infection. It should be noticed that the amounts of total proteins obtained from cytoplasmic fraction are generally four times more than proteins from the nuclear fraction extracted at each time point (Fig. 2C), yet equal amount of protein was used per lane for western blot based on the β-actin expression level constant in cytoplasm and nucleus (Fig. 2A and 2B) and similar level of background in each blot. Moreover, the redistribution of GAPDH upon JEV infection may happen at an early stage since the difference was observed promptly when protein was extracted immediately following virus infection (comparing Fig. 2A and 2B, lanes 1 and 7). Interestingly, the expression of nuclear-GAPDH re-increased at 12 h postinfection (Fig. 2B, lane 9) reaching to the comparable amounts at 18 and 24 h postinfection (Fig. 2B, lanes 10 and 11) and then decreased again at 36 h postinfection (Fig. 2B, lane 12). The differences were not observed in those uninfected cells (Fig. 2A). This result was consistent with the observation in the IFA (Fig. 1) that GAPDH was barely detected in the nucleus of infected cells at 36 hpi. Please note that there was a low molecular band, recognized by anti-GAPDH specific antibody, shown only in the nuclear fraction, which may be a derived and/or spliced product from the intact GAPDH. However, the functional relevance of the derived/spliced GAPDH remains to be investigated. Oppositely, the NS5 protein was detected in the cytoplasmic fraction at 12 h postinfection and throughout the infection period (Fig. 2B, lanes 3 to 6). Of particular interest, it was also detected in the nuclear fraction at 18 h through 36 h postinfection (Fig. 2B, lanes 10 to 12). Here we use β-tubulin, a cytoplasmic specific subcellular marker, to exclude the possibility of NS5 in the nuclear extract might having come from cytoplasmic contamination during fractionation. As shown in Fig. 2B, the β-tubulin was detected only in the cytoplasmic fraction but not in the nucleus fraction, indicating that the subcellular fractionation of nucleus versus cytoplasm was complete.

### Co-immunoprecipitation analysis of NS5 and GAPDH

**Figure 3:**
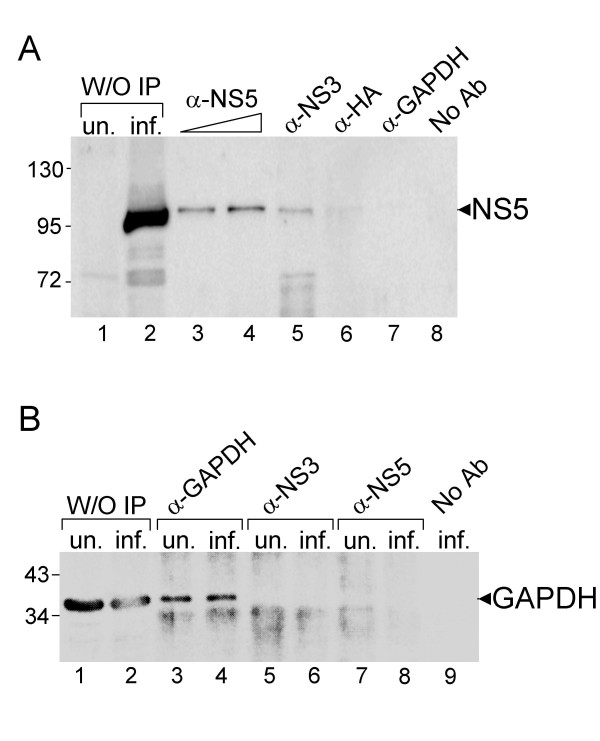
**No direct interaction of NS5 and GAPDH proteins was demonstrated by co-immunoprecipitation (Co-IP) followed by Western blot analyses against NS5 (A) or GAPDH (B)**. Lanes 1 and 2, 10 μg of uninfected (un.) and infected (inf.) cell extracts without (W/O) IP. Antibodies against various proteins for IP or no antibody added are indicated on the top.

In order to verify direct or indirect association of the NS5 protein with GAPDH in JEV-infected cells, we perform co-immunoprecipitation assays and Western Blot analysis. The NS5 was detected in dose-dependent manner with anti-NS5 antibody (Fig. 3A, lanes 3 and 4) or co-immunoprecipitated with anti-NS3 antibody (Fig. 3A, lane 5). However, the NS5 was not co-immunoprecipitated with anti-GAPDH antibody in the same assay condition (Fig. 3A, lane 7) neither with anti-HA antibody or no antibody (Fig. 3A, lanes 6 and 8). In addition, the GAPDH was co-immunoprecipitated with anti-GAPDH antibody either in uninfected or infected cell lysate (Fig. 3B, lanes 3 and 4) indicating that the antibody is suitable for immunoprecipitation. GAPDH was not co-immunoprecipitated with anti-NS3, anti-NS5 antibodies, or no antibody added (Fig. 3B, lanes 5–9). These results indicated that GAPDH does not directly interact with NS5 or NS3, suggesting that the host-viral protein colocalization may be via an RNA intermediate.

### GAPDH interacts with 3' ends of JEV RNAs in vitro

**Figure 4:**
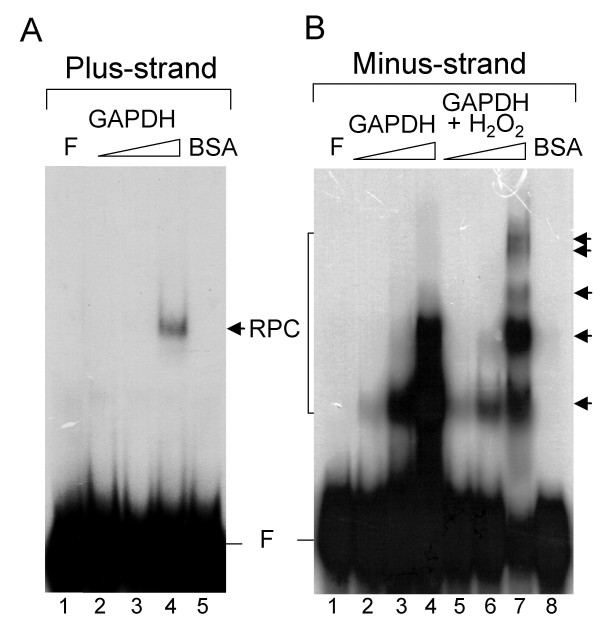
***In vitro *interaction of GAPDH with plus-strand (A) and minus-strand (B) of JEV riboprobes**. Lane 1, free probe (F). A. GAPDH (50 ng, 200 ng, 1 μg; lanes 2–4), or BSA (1 μg; lane 5) were added to the binding reaction as indicated on the top. B. GAPDH proteins (55 ng, 165 ng, 550 ng) without (lanes 2–4) or with 70 μM of H_2_O_2 _treated (lanes 5–7) were added to the binding reaction. RNA-protein complexes (RPC) are indicated by arrows.

The interactions of JEV NS5 as well as host factors with 3' stem-loop regions of plus- and minus-strands of JEV RNAs have been reported [4,22,23]. Here we ask whether the viral RNAs served as the intermediates for the colocalization of GAPDH and NS5. To address this issue, we use two viral RNAs: the 3' terminal 87 nts of plus strand (JEV(+)3'SL) and 3' end of minus strand corresponding to genome at nt 1–160 (JEV(-)1–160), for the *in vitro *gel mobility shift assays (EMSA). The initial approach was performing EMSA using cell extract mixed with the two riboprobes, respectively, followed by supershift assay with anti-GAPDH antibody. RNA-protein complexes were found indicating host proteins associated with the viral RNA, yet no supershift band was detected with the GAPDH specific antibodies (data not shown). This result may be due to the reason that GAPDH exists mainly as a tetramer comprising four identical 37-kDa subunits *in vivo *and is also known to form a variety of protein-protein complexes with other cellular proteins. These complexes may hinder accessibility of an epitope to the antibody we used as well as instabilities during gel electrophoresis. Alternatively, we chose purified GAPDH protein to perform EMSA and to characterize the interaction of GAPDH protein with plus- and minus-strands of viral RNAs. A mobility shift band was observed when plus-strand riboprobe incubated with 1 μg of purified GAPDH (Fig. 4A, lane 4) but was not detectable with amounts of 50 or 200 ng of GAPDH used (Fig. 4A, lanes 2 and 3). Addition of 1 μg bovine serum albumin (BSA) to the riboprobe resulted in no RPC band (Fig. 4A, lane 5), suggesting that the binding to GAPDH is specific. When JEV minus-strand riboprobe (JEV(-)1–160) was used, the PRC band was readily detected when GAPDH was as low as 55 ng (Fig. 4B, lane 2). At least two major PRC species at higher concentration of GAPDH (165 and 550 ng) were observed (Fig. 4B, lanes 3 and 4). It has been reported that oxidation of GAPDH enhances its binding to nucleic acids [24], thus 70 μM of H_2_O_2 _was added to the binding reaction. The intensity of the two major RPC species did not increase; however, three additional slow migration bands were observed when 550 ng of GAPDH was used (Fig. 4B, lane 7). Addition of 1 μg BSA did not bind to JEV(-)1–160 riboprobe indicating that the binding is specific (Fig. 4B, lane 8). These results indicated that GAPDH binds much more efficiently to the minus-strand riboprobe than to the plus strand.

## Discussion

GAPDH is an abundant and constantly expressed protein; it plays an essential role in glycolysis and many other important cellular functions [10,25]. In this study, we have shown that GAPDH colocalized with JEV NS5 in the infected cells (Fig. 1), adding that this colocalization may be due to its ubiquitous nature and relatively high abundance. Next, we demonstrated that the GAPDH and NS5 proteins did not interact directly (Fig. 3); instead, the interaction of these two proteins is through its RNA intermediates since the GAPDH binds to the 3' termini of plus- and minus-strand RNA of JEV (Fig. 4). Ribonucleoprotien complex in infected cells is involved in many specific RNA-protein and protein-protein interactions. It has been reported that NS3 and NS5 proteins of JEV and a cellular protein Mov 34 bind to the 3'-long stable hairpin of the plus strand [4,23]. We demonstrated that NS5 binds to the JEV(-)1–160 riboprobe (data not shown). Taken together, these results revealed that both NS5 and GAPDH bind to 3' termini of plus- and minus-strand RNAs of JEV, suggesting that the colocalization of these two proteins is due to both of them being bound to the viral terminal RNAs. Yet, similar or relatively close viral RNA regions responsible for both protein binding remain to be determined. Furthermore, our results indicated that GAPDH binds more efficiently to the minus-strand RNA than to the plus-strand RNA (Fig. 4). Recently, Wang and Nagy reported an interesting observation that GAPDH selectively binds to Tomato bushy stunt virus (TBSV) minus-strand RNA over plus-strand RNA [26], suggesting a model for promoting asymmetric RNA replication since they demonstrated that GAPDH bound an AUUUA pentamer sequence in the minus strand and retained minus strand RNAs in the replication complex [26,27]. Similarly, JEV(-)1–160 RNA sequence contains an UUUUA pentamer (complementary to nt 122 to 126 in JEV RP9 genome) which is located at the loop of the proximal stemloop of the minus strand. It is likely that GAPDH binds to this region. In addition, the interactions of GAPDH with secondary structures such as AU-rich helices RNA have been reported in various RNA viruses including human parainfluenza virus type 3 [28], hepatitis A and C viruses [29,30]. The overall AU contents of plus-strand riboprobe is about 50%, while the minus-strand riboprobe is about 60%, suggesting that the differences of AU contents may cause the selective binding of GAPDH. Whether such difference affects the plus and minus-strand synthesis of JEV RNA needs to be characterized; nevertheless, our results indicated that selective retention of the viral minus strand by GAPDH could be a common feature for the asymmetric replication of plus strand RNA viruses [27].

The role of GAPDH in several important nuclear functions has been identified in the past few years including participation in DNA replication/repair [31], regulation of histone genes expression [18], and modulation of telomere structure [19]. Furthermore, GAPDH interacts with microtubules and participates in nuclear membrane fusion [32]. Our results showed that nuclear-GAPDH is found at high levels in the uninfected cells (Fig. 2A) suggesting its normal function in the nucleus. In contrast, by infection with JEV, the nuclear-GAPDH appeared to be localized to the cytoplasm. It should be noted that overall expression of GAPDH in the uninfected and infected cells did not change significantly (data not shown), yet differences were found by subcellular fractionation of GAPDH (Fig. 2) indicating that JEV infection caused redistribution of GAPDH. Interestingly, the amount of nuclear-GAPDH decreased in early infection, increased between 12 to 24 h postinfection, and decreased again at 36 h postinfection. GAPDH is involved in the early stages of apoptosis, which trigger the translocation of GAPDH into the nucleus [17,33]. GAPDH contains nuclear localization signal (NLS) as well as nuclear export signal (NES) indicating that it shuttles between these two compartments and the translocation is reversible [34-36]. The redistribution pattern of the nuclear GAPDH indicated that it may play a signaling role in the apoptosis pathway during JEV infection. In addition, JEV NS5 was also detected in the nuclear fraction at 18 h through 36 h postinfection (Fig. 2B). Host proteins La, primarily a nuclear protein, and importin-beta were found to interact with the NS5 of Dengue virus suggesting that nuclear localization of NS5 may be via the assistance of these host proteins [37]. La protein also binds to NS3 as well as to the 5' and 3' ends of Dengue virus RNAs [38,39]. Recently, La protein was shown to bind to the 3'-SL of JEV [40]. These results indicate that nuclear localization of viral proteins may be essential for virus replication.

A low molecular band, recognized by the anti-GAPDH specific antibody, was detected only in the nuclear fraction (Fig. 2). GAPDH staining comprised of two bands was reported in the S49 cells and only the lower band was detected in the nuclear fraction by subfractionation on a sucrose gradient [41]. Observation of two bands apparently was due to using different subcellular fractionation methods since under the same condition, two bands were observed by subfractionation using sucrose gradients, while only one band was revealed using normal centrifugation [41]. Similarly, we detected only one GAPDH band from total cell lysate (Fig. 3B, lanes 1 and 2), while detecting two bands in the nuclear fraction prepared from the Nuclear/Cytosol Fractionation Kit (BioVision).

In summary, GAPDH interacts indirectly with JEV NS5 protein through the 3'-ends of the viral RNAs, resulting in redistribution of GAPDH in the infected cells. It is likely that the virus-induced redistribution of GAPDH is associated with the early stage of JEV replication/translation after it entered into the host cells. Results presented in this study support the notion that asymmetric replication of plus strand RNA viruses co-opt a host protein for the selective binding ability and ensure an optimal plus-strand/minus-strand viral RNA production. This is the first report to indicate redistribution of GAPDH correlates with JEV infection, which may open up additional avenues toward a deeper understanding of GAPDH regulating the replication of JEV.

## Competing interests

The authors declare that they have no competing interests.

## Authors' contributions

SHY designed and carried out the main experiments. MLL initiated the project. CFT performed the EMSA experiments and SJ. Chou constructed the plasmids. RYC conceived of the study, and participated in its design and coordination. All authors read and approved the final manuscript.
